# Prevalence and Correlates of Smoking and Readiness to Quit Smoking in People Living with HIV in Austria and Germany

**DOI:** 10.1371/journal.pone.0150553

**Published:** 2016-02-26

**Authors:** Helmut Brath, Igor Grabovac, Horst Schalk, Olaf Degen, Thomas E. Dorner

**Affiliations:** 1 Health Centre South, Vienna, Austria; 2 Institute of Occupational Medicine, University Clinic for Internal Medicine II, Medical University of Vienna, Vienna, Austria; 3 Schalk-Pichler Group Practice, Vienna, Austria; 4 University Clinic Hamburg-Eppendorf, Outpatient Centre, Hamburg, Germany; 5 Institute of Social Medicine, Centre for Public Health, Medical University of Vienna, Vienna, Austria; University of Florida, UNITED STATES

## Abstract

We aimed to investigate the prevalence and correlates of smoking in people living with HIV (PLWHIV) in Germany and Austria and their readiness to quit. A total of 447 consecutive patients with confirmed positive HIV status who were treated in different outpatient HIV centres in Austria and Germany were included. Nicotine dependence and stages of change were assessed by standardized questionnaires, and this was confirmed by measuring exhaled carbon monoxide. Prevalence of smoking was 49.4%. According to a multivariate logistic regression analysis, higher age (for each year of life OR = 0.96; 95% CI 0.92–1.00) and tertiary education level (OR = 0.43; 95% CI 0.15–0.79) were associated with a lower chance, and occasional (OR = 3.75; 95% CI 1.74–8.07) and daily smoking of the partner (OR 8.78; 95% CI 4.49–17.17) were significantly associated with a higher chance of smoking. Moderate (OR = 3.41; 95% CI = 1.30–9.05) and higher nicotine dependency level (OR = 3.40; 95% CI 1.46–7.94), were significantly associated with higher chance, and older age (for each year of life OR = 0.95; 95% CI = 0.91–0.99), with lower chance for readiness to quit smoking. Those results may be used to address preventive measures to quit smoking aimed at PLWHIV and the importance of addressing smoking habits.

## Introduction

Due to the effectiveness of antiretroviral therapy, HIV-related mortality has dramatically fallen, especially in industrialised countries, with the possible life expectancy of people living with HIV (PLWHIV) improving considerably [1, 2]. Today, HIV may be considered a chronic condition with new health issues emerging such as questions on healthy aging and an increase in non-AIDS related conditions such as different types of malignant or cardiovascular illnesses [3, 4]. It is estimated that in Germany there are around 80000 and around 9000 PLWHIV in Austria with both countries still not being able to gain a drop in the number of new diagnoses that have reached a plateau in the last few years [5, 6]. Health issues caused by smoking may have a severe effect on the quality of life of PLWHIV and should be appropriately addressed with prevention efforts and population oriented behavioural modification techniques [7].

Although it is the single most preventable cause of death, tobacco smoking remains one of the major public health concerns of today being associated with higher risk of cerebrovascular, pulmonary and malignant diseases [8, 9, 10]. In PLWHIV, smoking contributes significantly to an increase in mortality and morbidity [11, 12, 13].

A recent cohort study showed that in industrialised countries with good implementation of HIV therapy protocols, smoking causes twice as many years of life lost in PLWHIV than HIV alone [14]. Also, PLWHIV who smoke have a higher risk of infection in comparison to those who do not, as there is evidence to support the idea that smoking accelerates the decrease of CD4 cells [13], as well as having an attenuating effect on antiretroviral therapy [15]. In addition to the expected improvement in the clinical outcomes, PLWHIV who stop smoking significantly improved their quality of life which was apparent as early as after 3 months from the last smoked cigarette [16].

Although the literature supports the notion of great health benefits for PLWHIV who stop smoking, the prevalence in this population remains particularly high as there are a few smoking cessation programs aimed at PLWHIV [17, 18]. Survey done in the USA showed 2–3 times higher smoking rates among PLWHIV in comparison to the general public and other research done so far tend to support this data, especially pointing out high rates within the men who have sex with men (MSM) group who were also less likely to quit smoking compared to the general adult population [19, 20, 21]. This could be explained by the shared psychosocial factors in the MSM and PLWHIV groups such as social stigma, discrimination, higher depression prevalence, minority stress and limited health access [22, 23, 24]. Also, due to the physical changes occurring in PLWHIV a US group reported a close link between smoking and body image linking the success of smoking cessation to anxiety, stress, social support and depression [25]. A study from New York reported the prevalence of smoking at 59% in PLWHIV, out of these 50% were found to be moderately or severely addicted to nicotine. However, as high as 75% expressed the wish to stop smoking with 64% having tried to stop at least once during the past year [26]. In PLWHIV smoking status has been shown to change after the HIV diagnosis [27].

Smoking prevalence in the general public in German speaking countries is a point of great concern, especially in Austria where an increase in overall prevalence of smoking was found at 27% in men and 22% in women in 2014. Additionally, one fifth of the Austrian non-smoking population is exposed to second hand smoking [28]. In Germany, rates have decreased somewhat from 38.9% in men and 30.6% in women in 2000 to 32.6% in men and 26.9% in women as reported in 2013, however the rates also remain high in the younger adults [29, 30]. Smoking prevalence of PLWHIV in German speaking countries has so far not been investigated. The primary aim of this study was to assess the prevalence of smoking among PLWHIV in Germany and Austria who visit their physicians regularly, and to determine what characteristics could be associated with higher risks for smoking or continuing to smoke. Since it has been reported in previous studies, that smoking is associated with various socio-economic parameter and the amount of social support is associated with smoking and the change of smoking status [31, 32, 33] we included those factors in the assessment. Additionally we included the smoking status of the treating physician and smoking status of partners, since they have also been shown to be associated patients’ own smoking habits, willingness to quit or offering smoking therapy [34, 35].

## Participants and Methods

### Participants

Our study participants consisted of patients who visited seven different outpatient HIV treatment centres (Designated “A-G”) in Austria and Germany between 3^rd^ of January 2012 and 4th of October 2012.

To minimise inclusion bias study participants were chosen from consecutive patients that came for their regular visits to a clinic and were asked to participate in the study if they matched the inclusion criteria; at least 18 years old of both sexes, a known and serologically confirmed HIV diagnosis and a signed informed consent form. A total of 447 participants agreed to participate in the study.

### Methods

Our study was designed as a multi-centre cross-sectional study. Six of the study centres were located in urban areas of Germany and one in an urban Austrian setting. The participating centres were self-selected from the German and Austrian societies of medical doctors who work with HIV patients. Nine percent of the total membership from the German society and 30% from the Austrian society participated. Prerequisite for participation was a valid certificate for smoking cessation therapy by the German or the Austrian physician chamber.

After the consultation with the physician, each patient was asked to fill in a short 10 -minute questionnaire without the presence of the doctor. Smoking status was confirmed by measuring exhaled carbon monoxide (CO) levels. This test was administered by a qualified physician. The pseudonymised questionnaires were sent to the Medical University of Vienna for analysis. The link between patients and their identification number could only been done in the respective place where the data collection took place. The team working on the analysis had no information about the personal data of the participants.

The questionnaire used in our study consisted of four parts; general sociodemographic data and questions about current health, questions about smoking status and the Fagerström Test for Nicotine Dependence (FTND) to assess the levels of dependence, and Readiness to Change Smoking Behaviour questionnaire used to determine the willingness to stop smoking.

A total of 13 questions about sociodemographic, financial and individual characteristics of the participants were included in the questionnaire (age, sex, sexual orientation, education level, relationship status, smoking status of the partner, stage of HIV infection, antiretroviral and antidepressant therapy, IV-drug use, history of cardiovascular disease and cancer and knowledge of the smoking status of the chosen physician as seen in Table 1). It consisted of both multiple choice answers as well as questions with free answers. Smoking status was assessed with the question: ‘Did you smoke at least one cigarette within the last 7 days?’. If this was answered with ‘Yes’, they were further asked if they smoked daily, if they changed smoking habits after the HIV diagnosis, if they were exposed to cigarette smoke at their workplace and about the quantity of daily consumed cigarettes, and wether they have a wish to quit smoking or not.

**Table 1:** Sociodemographic and medical data of smokers and non-smokers.

	Total	Smokers	Non smokers	p
N	447	221	222	
Age (Years, mean value)	45.5	43.2	47.8	<0.001
Sex % (N)				0.042
Male	86.4 (386)	88.2 (195)	84.2 (186)	
Female	11.2 (50)	8.1 (18)	14.4 (32)	
Missing	2.5 (11)	3.6 (8)	1.4 (3)	
Sexual Orientation % (N)				0.445
Homosexual	66.2 (296)	69.7 (154)	62.6 (139)	
Bisexual	6.7 (30)	5.4 (12)	26.1 (58)	
Heterosexual	23.9 (107)	22.2 (49)	7.7 (17)	
Missing	3.1 (14)	2.7 (6)	3.6 (8)	
Educational level % (N)				0.096
Primary	13.2 (59)	15.4 (34)	11.3 (25)	
Secondary	56.4 (252)	59.3 (131)	54.5 (121)	
Tertiary	25.5 (114)	19.9 (44)	29.7 (66)	
Missing	4.9 (22)	5.4 (12)	4.5 (10)	
Relationship status % (N)				0.062
In a relationship	56.0 (250)	55.7 (123)	56.3 (125)	
Not in a relationship	36.8 (165)	39.8 (88)	33.8 (75)	
Missing	7.2 (32)	4.5 (10)	9.9 (22)	
Stage of HIV Infection^a^ % (N)				0.627
A	56.8 (254)	56.6 (125)	56.8 (126)	
B	22.6 (101)	24.9 (55)	20.3 (45)	
C	19.0 (85)	17.2 (38)	21.2 (47)	
Missing	1.6 (7)	1.4 (3)	1.8 (4)	
Antiretroviral therapy: Yes. % (N)	85.3 (381)	82.2 (182)	88.0 (195)	0.104
Antidepressant therapy: Yes. % (N)	16.0 (71)	14.0 (31)	18.0 (40)	0.321
IV- drug use: Yes. % (N)	4.1 (18)	6.9 (15)	1.4 (3)	0.004
Cardiovascular disease: Yes. % (N)	12.4 (55)	10.2 (22)	14.7 (33)	0.161
Cancer: Yes. % (N)	3.8 (17)	3.2 (7)	4.6 (10)	0.470
Smoking status of the partner^b^: % (N)				<0.001
Smokes daily	39.4 (176)	58.5 (129)	20.8 (46)	
Smokes sometimes	14.3 (64)	16.3 (36)	12.0 (27)	
Doesn’t smoke	45.4 (203)	24.4 (54)	67.2 (149)	
Missing	0.8 (4)	0.8 (2)	0.0 (0)	
Smoking status of the chosen physician: Yes, % (N)				0.478
Physician smokes	6.5 (29)	7.7 (17)	5.4 (12)	
Physician doesn’t smoke	36.0 (161)	37.6 (83)	34.2 (76)	
Don’t know	55.0 (246)	52.0 (115)	58.6 (130)	
Missing	2.5 (11)	2.7 (6)	1.8 (5)	

^a^ The stage of disease is based on CDC’s Surveillance case definition for HIV infection [44].

^b^ Only those participants who indicated that they have a partner

The Fagerström Test for Nicotine Dependence (FTND) is a standardized questionnaire used to assess the level of nicotine dependence in the participants who smoke. It consists of 6 statements; 2 of them are a Likert-type scale ranging from 0 to 3. First of the Likert type questions asks about the time passed until the first cigarette of the day, while the second estimates how many cigarettes a day the person consumes. Other 4 questions are “Yes/No questions” where a score of 1 is given for “Yes” and 0 to “No” answers. Therefore, the result is a numerical sum of the answers given and can range between 0 and 10 [36]. For this study the German language version of the FTND was used that has been used in different studies [37]. The results of the Fagerström tests were summarised into three categories based on dependency level (three categories, high, medium and low) with the respective cut-off values of 0–2, 3–4 and 5 and more points scored on the FTND [38].

Readiness to change smoking behaviour (Fragebogen zur Änderungsbereitschaft bei Rauchern (FÄR)) is based on the stages of change (SOC) trans-theoretical model developed by Prochaska and DiClemente. SOC describes 4 stages which a person passes in order to resolve an addictive problem starting at “precontemplation” (person is not planning and is unaware of the need to change) and continuing to “contemplation” (characterised by ambivalence and weighing between the “pros” and “cons” of changing their behaviour), “action” (people are in the process of change, need to strengthen their commitments) and finishing with “maintenance” (change is complete it is necessary to be aware of the need to fight the urges that may lead back to unhealthy behaviour). This is a cyclic model with one stage going into the next with the relapsers re-entering the cycle again at the “precontemplation” or “contemplation” stages. It might take several cycles until a person finally resolves the addictive problem. The FÄR offers 12 statements on which a person states the level of agreement with the statement on a 5 point scale with 1 being “not agree” and 5 “agree” respectively, and it corresponds to the German language version of the SOC. Scoring is based on the sum of the items that range from -2 to 2 points (after recoding the 1–5 point scale) with the sum total ranging from -8 to 8 points. Each of the stage is presented as a subscale within the questionnaire and the participants were allocated to one of the stages based on the subscale in which they achieved the highest (most positive value) score. Items were averaged to form a mean value, which means that a higher score on this scale represented a higher level of readiness to change the behaviour [39, 40].

Exhaled CO levels were measured by the “Micro + Bedfont Smokerlyzer with Colour Touchscreen” manufactured by “Bedofont Scientific Ltd” which was shown to have a good predictive value for smoking detection. The smokerlyser was calibrated, used and stored according to the manufacturer’s instructions. Considering that in centre “A” only smoker CO levels were measured, this centre was not included in the analysis of CO levels. Participants who had CO levels higher than 6 ppm were presumed to be smokers [41, 42, 43].

Descriptive statistics were applied for each variable. Quantitative variables were reported as mean values and standard deviation in the case of normal distribution, and qualitative variables were reported as frequency and percentage. Differences in frequency of certain categorical variables were determined by the Chi^2^ –test, and where appropriate to determine the differences of mean values the T-test (for unpaired samples) was used. Binary multivariate logistical regression analyses were performed in order to assess which characteristics were associated with current smoking as well as the desire to quit smoking (Tables 2 and 3). Variables (age, sex, IV-drug use, cardiovascular disease, education level, use of antiretroviral therapy, relationship status, smoking status of the partner) for the final multivariate model were chosen on the basis of the p<0.2 cut off point. All p-values below 0.05 were considered as statistically significant. The statistical analysis was performed using the SPSS 21.0 statistical software (Chicago, IL, www.spss.com).

**Table 2:** Smoking characteristics of participants who smoke (N = 221).

Variable	Answer	% (N)
Daily smoking	Yes	87.3 (193)
	No	12.2 (27)
	Missing	0.5% (1)
Number of cigarettes a day	Up to 10	24 (53)
	11–20	37.1 (82)
	21–30	22.2 (49)
	31 and more	13.1 (29)
	Missing	3.6 (8)
Changes in smoking after HIV diagnosis		
	Reduced	15.8 (35)
	Increased	15.4 (34)
	Didn't change	68.3 (151)
	Missing	0.5 (1)
FTND Category	Low dependency	25.3 (56)
	Moderate dependency	24.9 (55)
	High dependency	47.1 (104)
	Missing	2.7 (6)
FAR Stage	Precontemplation	15.4 (34)
	Contemplation	48.4 (107)
	Preparation	15.4 (34)
	Action	10.0 (22)
	Unclassified	7.7 (17)
	Missing	3.2 (7)
Do you wish to quit smoking?	Yes	56.1 (124)
	No	35.7 (79)
	Missing	8.1 (18)
CO level (ppm, mean value)	16.9	

**Table 3:** Multivariate logistic regression model of characteristics associated with smoking in the past 7 days (N = 447).

Variable	OR	95% CI	P
Age (each year)	0.94	0.91–0.97	<0.001
Sex (Ref: female)			
Male	3.50	1.44–8.51	0.006
Relationship status (Ref: not in a relationship)			
In a relationship	1.48	0.66–3.34	0.336
Missing	0.39	0.12–1.24	0.111
Smoking status of the partner (Ref: doesn’t smoke)			
Smokes sometimes	3.75	1.74–8.07	0.001
Smokes daily	8.78	4.49–17.17	<0.001
No partner	10.12	4.19–24.40	<0.001
Education level (Ref: primary)			
Secondary	0.74	0.35–1.55	0.422
Tertiary	0.43	0.15–0.79	0.013
Missing	1.91	0.42–8.60	0.399
IV- Drug use (Ref: no)			
Yes	3.63	0.89–14.87	0.073
Cardiovascular disease (Ref: no)			
Yes	1.15	0.53–2.50	0.716
Antiretroviral medication (Ref: no)			
Yes	0.55	0.27–1.09	0.089

### Ethical Considerations

This study was approved by the Ethics Commissions of the City of Vienna (EK 11-115-VK_NZ) for the centre in Vienna, and the Ethics Commission of the Doctor’s Chamber Hamburg (PV3965) for the centres in Germany.

## Results

In total, data was collected from 447 participants with the response rate of 92%, ranging from 69.1% to 98.0% in different collection centres. In the participating centres there were significant differences in the proportion of MSM, female and male participants and educational level of the participants. Proportion of MSM in PLWHIV ranged from 14% to 34%, 0% to 28% for female and 6.5% to 25.1% for male participants. Significant differences in educational level were observed for primary level (elementary school) 6.8% to 22%, secondary (highschool) 4.0% to 23% and tertiary level (university) 3.5% to 27.2%.

Sociodemographic data and medical history from the participants, smokers and non-smokers, is shown in Table 1.

### Smoking history

Two hundred twenty one or 49.4% of our participants indicated that they had smoked in the last 7 days and were therefore designated as smokers for the purpose of this study. A further 222 or 49.7% said that they did not smoke in the last 7 days and were therefore designated as non-smokers. The mean value of CO in exhaled air was 11.2 (SD:10.5) ppm with a range from 0–55 ppm. Significantly more smokers than non-smokers had higher values of CO in exhaled air (16.9 vs 4.5 ppm; p<0.001).

Based on our cut off value of 6 ppm, we found that 14% of non-smokers had higher values than 6 ppm, as well as 14% of smokers had lower levels than 6 ppm.

A total of 298 (66.7%) participants said that they had ever smoked daily while 133 (29.8%) participants stated that they had never smoked. Two hundred and ninety-five participants or 66.0% stated that they are never or almost never exposed to second-hand tobacco smoke at their workplace, while 66 (14.8%) estimate their exposure to less than an hour and 26 (5.8%) to a five hours and a further 29 (6.5%) for more than five hours a day.

In addition, in the binary multivariate analysis smokers were significantly more likely to be younger (M = 43.2, p< 0.001) male (88.2%, p = 0.042) and were more frequent IV-drug user (6.9%, p = 0.004) compared with non-smokers. Subjects whose partners smoked, were significantly more likely to smoke themselves compared to those whose partners did not smoke (58.5% vs 20.8%, p<0.001). No significant differences between smokers and non-smokers were found with respect to sexual orientation, education, relationship status, CDC stage, treatment with antiretroviral or anti-depressive therapy, history of cardiovascular disease or cancer and smoking habits of the attending physician (Table 1).

### Information on smoking habits of smokers

In the smoking population 193 (87.3%) participants said that they smoked daily with most participants (37.1%) smoking between 11–20 and up to 10 cigarettes a day (24.0%). Since the HIV diagnosis 35 (15.8%) participants have made reductions in their cigarette consumption while 34 (15.4%) have increased it and 151 (68.3%) participants claimed that their smoking habits haven’t changed after the diagnosis (Table 2). One hundred and ninety six or 51.1% participants who smoke estimated they had no exposition to cigarette smoke at the workplace and 44 (19.9%) estimated being exposed less than an hour, while 18 (18.1%) estimated an exposition of 1–5 hours and 23 (10.4%) for more than 5 hours a day.

### Scores on the Fagerström Test for Nicotine Dependence

The mean score on the FTND was 4.3 (SD:2.7). Fifty six (26.0%) of our participants were classified as having a low dependence level, a further 55 (25.6%) were classified as moderately dependent and the rest of 104 (48.4%) were classified as highly dependent on nicotine (Table 2).

### Scores on Readiness to Change Smoking Behaviour

One hundred and twenty four (56.1%) of the smokers responded that they wanted to quit smoking, 79 (35.7%) responded “No” and a further 18 (8.1%) did not answer the question. According to FÄR, 34 (15.4%) could be classified as being in the “precontemplation” stage, 107 (48.4%) in “contemplation”, 34 (15.4%) in “preparation”, and 22 (10.0%) in the “action” stage, respectively. Eleven percent of the respondents could not be clearly assigned to a stage or did not answer this part of the questionnaire (Table 2).

### Sample characteristics and their association with smoking and readiness to quit smoking

From the multivariate logistic regression analysis we see that older age (for each year of life OR = 0.96; 95% CI 0.92–1.00) and tertiary (university) education level (OR = 0.43; 95% CI 0.15–0.79) was associated with a lower chance of smoking, male sex (OR = 3.50; 95% CI 1.44–8.51) and especially the smoking status of the partner (OR 8.78; 95% CI 4.49–17.17) proved to be significantly associated with a higher chance of smoking (Table 3). Moderate (OR = 3.41; 95% CI = 1.30–9.05) and higher nicotine dependency level (OR = 3.40; 95% CI 1.46–7.94), were significantly associated with a higher readiness to quit smoking, and older age (for each year of life OR = 0.95; 95% CI = 0.91–0.99) with a lower readiness to quit (Table 4).

**Table 4:** Multivariate logistic regression model of characteristics associated with readiness to stop smoking among current smokers (N = 221).

Variable	OR	95% CI	p
Age (each year)	0.95	0.91–0.99	0.015
Sex (Ref: female)			
Male	0.76	0.19–3.06	0.697
Relationship status (Ref: not in a relationship)			
In a relationship	1.44	0.35–6.05	0.613
Missing	2.14	0.29–16.14	0.458
Smoking status of the partner (Ref: doesn’t smoke)			
Smokes sometimes	0.48	0.13–1.76	0.270
Smokes daily	0.43	0.14–1.28	0.131
No partner	0.94	0.20–4.39	0.939
Education level (Ref: primary)			
Secondary	2.24	0.82–6.14	0.117
Tertiary	1.63	0.50–5.29	0.412
Missing	0.21	0.02–1.85	0.161
Nicotine Dependence (Ref: low dependence)			
Moderate dependence	3.42	1.30–9.05	0.013
High dependence	3.40	1.46–7.94	0.005
IV- Drugs (Ref: no)			
Yes	1.80	0.39–8.33	0.448
Cardiovascular disease (Ref: no)			
Yes	2.70	0.79–9.23	0.111
Antiretroviral medication (Ref: No)			
Yes	1.29	0.50–3.30	0.589

## Discussion

Our results show the prevalence of smoking in PLWHIV population in outpatient HIV treatment centres in Austria and Germany to be at 49.4%, almost twice as high than the rates found in the general public (27% in men and 22% in women in Austria and 32.6% in men and 26.9% in women in Germany), and supports other findings from similar studies that showed higher prevalence rates in PLWHIV from 37–70% with the rates going even higher in lower socioeconomic groups [14, 18, 20, 26, 31, 45]. Our prevalence levels corresponded well with measured exhaled CO levels. At the mean value of 11.2 (SD ±10.5) ppm, values were found significantly higher in smokers than in non-smokers as seen in previously reported studies (Table 1) [43]. Also, based on our cut off value of 6 ppm, 14% of those that indicated that they do not smoke had higher values than the cut off value, and 14% of those that reported being smokers had values lower than the cut off value. This may be explained by either false response given in the survey or that some participants have smoked in the last seven days but not in the last 24 hours.

Since the HIV diagnosis, the same number of participants reduced and increased their cigarette intake. The found rate of the participants who lowered their smoking intake is low compared to other studies showing rates of post-diagnosis quitting from 20 to 49% [27, 46]. This clearly indicates that there is more need to educate health care professionals who work with PLWHIV to better motivate (as the same number of participants reduced and increased cigarette intake) their patients in accepting healthier lifestyles, especially as research indicates that a number of malignant diseases are preventable in PLWHIV if they stop smoking [18].

Also, smokers were significantly more likely male and had more frequently a history of IV-drug use than non-smokers, which was also found in the literature and in line with the general population [31, 45]. No other significant differences between smokers and non-smokers were found based on other characteristics which might be in connection with the relatively small sample size. In contrast to other studies that found a high percentage of smokers in MSM [19, 20, 21], we did not find a significant association of smoking status with sexual orientation, with a high prevalence of smoking disregarding the sexual orientation.

The noted higher prevalence of substance abuse (smoking and IV-drug use) might be associated with coping strategies of an HIV seropositive status. An investigation by Webb et al. showed that people who had less social support and experience more stress were found to smoke more. This could be linked to the fact that smoking may be a source of support for PLWHIV [47]. A pilot study found that men who were HIV positive did understand the adverse health effects that smoking has, however were not interested in quitting as they felt it increased their well-being and felt that they were going to die before the adverse health effects of smoking could take place [48].

It is worth noting that although not found statistically significant, more PLWHIV who were smokers knew about the smoking status of their physicians.

The majority of our participants were highly nicotine dependent, which is similar to other published results. [26, 49]. However, this proportion is higher than the one in the general Austrian population, where around 36% were found to be highly nicotine addicted smokers [50]. Unfortunately, higher dependence levels are associated with lesser resolve to stop smoking [45, 51]. This was shown in the multiple regression model where strongest association for readiness to quit smoking was found with higher education levels and lower nicotine dependency, which was also shown in other research [45].

A clear majority of our smoking participants responded that they wanted to quit smoking. However, according to the FÄR, the majority of the participants were in the “contemplation” phase, and only 10% are found to be in the “action” phase. Although the biggest proportion was in the “contemplation” phase it is still encouraging, as it shows that with work that focuses on strengthening their resolve and giving more of an emphasis on positive aspects of accepting healthier behaviour patterns may encourage the change [26, 49, 52].

We found a significant association for smoking behaviour with lower age, male sex, lower education level and especially the smoking status of the partner, all characteristics that have been shown to influence smoking habits [53, 54]. Although some of them have been reported numerous times (such as higher prevalence of smoking in men and people with lower socioeconomic status), our results showed that certain social factors play a role in smoking behaviour. PLWHIV who have partners who smoke regularly or sometimes were more often found to smoke themselves. A recent study from Germany also showed that smokers with non-smoking partners are better motivated and eager to quit smoking in comparison to those with partners who smoke [55]. Other research also noted the importance of social networks for smoking cessation in PLWHIV. Reynolds reported that in their study all PLWHIV who smoked had partners who smoke as well [48]. Therefore, physicians and other health professionals should address smoking status of the partner when conducting smoking cessation programmes.

It is interesting to note that a large proportion of PLWHIV were exposed to second hand smoke on their workplace. In our study, this was even higher compared to the also high proportion in the general Austrian population [28]. Reason for this is probably the fact that still, public health efforts to protect people from smoking, are lagging behind European standards [56]. In line with this is the fact that in the Austrian general population the proportion of smokers is still increasing [28].

The limitations of the study are the overrepresentation of male participants. However, this represents the PLWHIV community that is treated in the extramural setting in Austria and Germany, very well [57]. Since smoking behaviour remains higher in males than in females this could show a higher prevalence level in PLWHIV. On the other side, the larger proportion of higher educated participants as well as the possibility that the participants gave more socially desirable answers, perhaps wanting to please their physicians, could contribute to some data distortions as well. Due to the fact that we used CO levels as a biomarker for smoking, a social desirability bias should not affect the prevalence of smoking, however we cannot exclude that this has an effect on readiness to quit and the calculated SOC.

Our results showed that the prevalence of smoking within the PLWHIV population in HIV treatment centres in Germany and Austria is higher than the general population in concordance with previously published research on this topic. Having reported the correlates of smoking we believe that the presented result could be used to motivate health care workers working with PLWHIV to better inform their patients about the health benefits of smoking cessation as well as creating better tailored programs and public health initiatives to raise awareness of this issues within the PLWHIV community.

## Supporting Information

S1 Dataset Dataset from the Medical University of Vienna. (SAV)
